# Altered glucose disposition and insulin sensitivity in peri-pubertal first-degree relatives of women with polycystic ovary syndrome

**DOI:** 10.1186/1687-9856-2012-14

**Published:** 2012-05-29

**Authors:** Nouhad Raissouni, Andrey Kolesnikov, Radhika Purushothaman, Sunil Sinha, Sonal Bhandari, Amrit Bhangoo, Shahid Malik, Revi Mathew, Jean-Patrice Baillargeon, Maria Isabel Hernandez, Michael Rosenbaum, Svetlana Ten, David Geller

**Affiliations:** 1 Division of Pediatric Endocrinology at Infants and Children's Hospital of Brooklyn at Maimonides & SUNY Downstate Medical Center, 1068 48th St, Brooklyn, NY, 11219, USA; 2 Department of Pediatrics, Division of Pediatric Endocrinology, Vanderbilt University School of Medicine, Monroe Carell Jr. Children's Hospital at Vanderbilt, 11134A Doctors' Office Tower, 2200 Children's Way, Nashville, TN, 37232-9170, USA; 3 Department of Medicine, and Physiology and Biophysics, Division of Endocrinology, University of Sherbrooke, 3001, 12e Avenue Nord, Sherbrooke, QC, J1H 5N4, Canada; 4 Departments of Pediatrics, Endocrinologa Infantil, Instituto de Investigaciones Materno Infantil (IDIMI), Universidad de Chile, Clinica Las Condes Santiago, Chile; 5 Department of Pediatrics, Division of Pediatric Endocrinology, Children's Hospital of New York-Presbyterian, 622 West 168th Street, PH-5E-522, New York, NY, 10032, USA; 6 Department of Pediatrics, Division of Pediatric Endocrinology, Cedars-Sinai Medical Center, David Geffen- University of California, Los Angeles School of Medicine, 8700 Beverly Blvd. Room 4220 North Tower, Los Angeles, CA, 90048, USA

**Keywords:** PCOS, Insulin resistance, Insulin sensitivity, Anovulation, Hyperandrogenemia, Premature pubarche, Diabetes mellitus, Beta cell function

## Abstract

**Background:**

First-degree relatives (FDRs) of women with PCOS are at increased risk for impaired insulin sensitivity and diabetes mellitus. Glucose tolerant FDR have evidence of insulin resistance and hyperinsulinemia prior to emergence of frank PCOS.

**Aim:**

To study insulin dynamics parameters in the early adolescent FDR of women with PCOS.

**Methods:**

This is a cross-sectional study involving 18 adolescents whose mothers or sisters had been diagnosed with PCOS and 21 healthy, age-matched control adolescents without FDR. Subjects underwent anthropometric measurements, steroid profiling and frequently sampled Intravenous Glucose Tolerance Test (IVGTT), Homeostasis Model Assessment (HOMA) index, Glucose Disposal Index (GDI), Acute Insulin Response (AIR) and Quantitative insulin sensitivity check index (QUICKI) were derived from IVGTT results.

**Results:**

FDRs showed significantly higher mean HOMA and lower GDI. There were no differences in mean age or BMI Z-score between the cohorts. No differences in sex steroids or AIR were identified between groups.

**Conclusion:**

Female adolescent FDR of women with PCOS have higher HOMA index and lower QUICKI, reflecting altered insulin sensitivity and lower GDI reflecting poorer beta-cell function. The presence of multiple risk factors for type 2 diabetes suggests that aggressive screening of the early adolescent FDR of women with PCOS is indicated.

## Introduction

POLYCYSTIC OVARY SYNDROME (PCOS) is an endocrine-metabolic disorder, which is highly prevalent (5–10%) in reproductive-age women [1-3]. PCOS is characterized by hyperandrogenism [4,5]. The phenotypic characterization is heterogeneous; PCOS can manifest in the prepubertal years as premature pubarche [6]; hirsutism, acne and anovulatory cycles may remain clinically silent until late adolescence. Peripheral insulin resistance plays a key role in the pathogenesis of this syndrome. It has been suggested that insulin excess facilitates ovarian and/or adrenal hyperandrogenism [7-10]. Insulin resistance in PCOS women has long-term health consequences, predisposing to type 2 diabetes mellitus, cardiovascular disease and pregnancy-associated disorders like infertility, miscarriage, premature labor and gestational diabetes [11-15].

Female first-degree relatives (FDRs) of PCOS-affected women are at higher risk for developing PCOS symptoms [16]. FDRs are also at higher risk of developing the endocrine and metabolic co-morbidities of PCOS, such as obesity, insulin resistance and impaired insulin sensitivity, hyperlipidemia and metabolic syndrome[17-21]. An abundant literature supports familial clustering of hyperandrogenemia in PCOS women, consistent with a genetic contribution to the disease [16,22]. Recent studies have shown an increased prevalence of hyperandrogenism and insulin resistance in adult FDRs of PCOS [16].

Despite the alarming increase in the prevalence of type 2 diabetes in children, and efforts to identify risk factors for the development of this disease in children studies of glucose homeostasis in pediatric FDRs of PCOS, have not been performed. In the present study we assessed the insulin secretion (β-cell function), insulin sensitivity, adrenal and ovarian steroid levels in peri-pubertal daughters and sisters of women diagnosed with PCOS. Our purpose was to determine the presence of early biochemical changes in females at risk of PCOS before clinical manifestations occurred and to evaluate whether insulin resistance or hyperandrogenemia occurs before in FDRs of PCOS women. We performed IVGTT to more comprehensively study the insulin dynamics in this cohort, and compared the results with those of an age- and weight-matched group of controls who were daughters or sisters of women without PCOS. The short version of IVGTT provides enough testing points to calculate indexes to assess insulin sensitivity and β-cell function. We also compared the steroid levels in both groups to evaluate the existence of the hormonal imbalance in FDRs. We hypothesized that being an FDR of a PCOS conveys an independent risk for the development of type 2 diabetes independent of other biochemical or clinical evidence of PCOS.

## Subjects and methods

### Study subjects

We studied 18 healthy premenarchal girls (mean age of 11.6 ± 1.1 years, range 8–14) whose mothers or sisters had been diagnosed with PCOS and were followed in adult and pediatric endocrinology clinics at Maimonides Medical Center, University of Sherbooke, University of Chile, Vanderbilt University and Cedars-Sinai Medical Center. All had been diagnosed with PCOS based upon the National Institute of Child Health and Human Development criteria for PCOS [23], including history of documented chronic oligomenorrhea or amenorrhea and hyperandrogenism, with the exclusion of secondary causes. The anthropometric, insulin dynamics and steroid data of FDR subjects was determined for NIH K23 HD040325, “Insulin Resistance in Adolescents at High Risk for Polycystic Ovary Syndrome".

The control group consisted of 21 healthy premenarchal girls (mean age of 12.1 ± 0.4 years, range 8–14) whose mothers had no history of irregular menstrual cycles or hirsutism. Children were recruited from a school-based study that is part of Reduce Obesity and Diabetes (ROAD) Project, a collaborative project between Maimonides Medical Center and Columbia University Medical Center, Cohen Children’s Medical Center, Mount Sinai Medical Center, Winthrop University Hospital and New York City public schools. This ongoing project is supported by Academy for Medical Development and Collaboration (AMDeC). These children had no personal history of diabetes or family history of irregular menstruation, diabetes or hirsutism.

First-degree relatives of PCOS women were recruited and tested following approval from the Institutional Review Boards at Cedars-Sinai Medical Center (Los Angeles), Sherbrooke University (Sherbrooke, Canada), University of Chile (Santiago, Chile), Vanderbilt University (Nashville, TN), and Maimonides Medical Center (Brooklyn, New York). The ROAD Project study was approved by the Institutional Review Board for each participating hospital, school boards and the Department of Health and Education. Written informed consent was obtained from all parents and assent from the peri-adolescent study subjects.

## Study protocol

### Assessment

All study subjects were either tested at Maimonides Medical Center Clinic (MMCC), ROAD school based study or at Cedars-Sinai Medical Center, Vanderbilt University, University of Sherbrooke or University of Chile. Enrollees presented after an overnight fast. A comprehensive medical assessment and medical history were obtained, including a personal and family history of type 2 diabetes, metabolic syndrome or heart disease. Anthropometric measurements included weight, height, body mass index (BMI), and waist circumference (waist: midway between the lower rib margin and the iliac crest). Age- and sex-specific BMI z-scores were calculated by using National Center for Health Statistics (NCHS) data [24]. Body fat composition was determined by bioelectrical impedance (Body Fat Analyzer, Model HBF-306, Omron, Gays Mills, WI). A Tanner stage was assigned to each study subject based on levels of both DHEAS and E2. No participants in the study were taking medications known to affect either sex steroids or carbohydrate metabolism.

#### IVGTT

Rapid frequently sampled intravenous glucose tolerance testing (IVGTT) was performed on all FDRs [25]. Each subject was given IV 25% Dextrose at 2 ml/Kg (max 25gm of Dextrose) delivered over 1 min, and blood was drawn through the same butterfly needle for measurements of serum glucose and insulin at 2, 3, 4 and 5 min after glucose administration. Short version of IVGTT (5 point over 5 minutes versus 3 hr classic IVGTT) had been chosen in control group because it was less time consuming in the school-based study.

#### Hormonal assay

Additional baseline blood samples were obtained for determination of DHEAS and Estradiol (E2).

Serum glucose was determined by the glucose hexokinase procedure from Raichem. The intra- and interassay coefficient of variation of this method was less than 3%. Serum insulin was determined by a solid phase sandwich immunoassay developed by Wallace/Perkin-Elmer. The intra-assay coefficient of variation of this method is 3-6%. Serum assays for DHEAS and E2 were performed by Labcorp Institute (Burlington, NC). Assay sensitivities for DHEAS and E2 were 1.7 ng/dl, 10 pg/ml, respectively. Intra- and interassay coefficients of variation were 7.9-9.8% for DHEAS and 97% for E2.

### Calculations

Insulin resistance was estimated by the Homeostasis Model Assessment for Insulin Resistance (HOMA-IR), calculated as [fasting insulin (μU/ml) x fasting glucose (mg/dl)]/405] [26] and insulin sensitivity was estimated by Quantitative Insulin Sensitivity Check Index (QUICKI), calculated as [1/(log fasting insulin μU/ml) + log (fasting glucose mg/dl)] [27]. The acute insulin response (AIRg) was calculated as mean incremental rise in plasma insulin at 3 and 5 min after IV glucose. Pancreatic β**-**cell function was assessed by calculating the Glucose Disposal index as [log_10_ (AIRg x fasting glucose concentration/fasting insulin concentration)] [25,28].

### Statistical methods and analysis

Data are expressed as mean ± SD. Freidman’s repeated measures ANOVA was used to compare variables (age, BMI Z-score, HOMA, QUICKI, GDI, DHEAS, etc.) within the same group. Comparisons of means between the PCOS affected first-degree relatives group (PCOS-FDR) and the control group were performed using the Unequal Variance, Unequal Sample Size *t*-test. Regression analysis and Spearman correlations were used to evaluate the relationship between the variables of interest. Statistical analysis was performed using SPSS Statistics 17.0.

## Results

### Anthropometry and hormonal assay

There were no significant differences between the PCOS-FDR and the control groups with respect to age, BMI, BMI Z score, waist circumference or percent of body fat (Table 1). There were no significant differences in serum DHEA-S between groups. E_2_ levels were significantly higher in PCOS-FDR group comparing to the control group. Both groups had similar Tanner stage distributions, as a function of DHEA-S and E2 levels.

**Table 1:** Anthropometric characteristic of PCOS FDR and Control Daughters (Cd)

	**FDR-PCOS (n = 18)**	**Cd group (n = 21)**
**Age (yr)**	**11.6 ± 1.4**	**12. ± 0.8**
**BMI (kg/m**^**2**^**)**	**21.5 ± 3.5**	**21.5 ± 3.2**
**BMI Z Score**	**1.01 ±0.85**	**0.93 ± 0.9**
**Waist Circumference (cm)**	**72 ± 10.9**	**76 ±8.7**
**Body Fat %**	**25 ± 2.5**	**25.76 ± 3.2**

Data are presented as the mean ± SD. *P value <0.05.

### IVGTT

The HOMA-IR ratio was significantly higher, and both the QUICKI and GDI parameters were significantly lower in the PCOS-FDR group compared to the control group (Table 2). The HOMA-IR ratio findings were unchanged even after segregating the PCOS-FDR and control groups according to Tanner stage. AIR was not significantly different between groups.

**Table 2:** Biochemical characteristics in FDR-PCOS and Control Daughters (Cd) groups

	**FDR-PCOS Group (n = 18)**	**Cd Group (n = 21)**
**DHEA-S**	**63 (65)**	**99.5 (71)**
**AIRg**	**80 (54)**	**97 (43)**
**QUICKI**	0.32 (0.03) *	**0.35(0.02)**
**HOMA-IR**	3.45 (1.7)*	**2.04 (1.6)**
**GDI**	2.6 (0.46) *	**2.98 (0.27)**

Data are presented as the mean ± SD. *P value <0.05.

## Discussion

The major findings of this study are that both decreased insulin sensitivity and beta-cell function are evident in premenarachal peripubertal female FDR’s of PCOS without clinical or biochemical evidence of PCOS. These data suggest that having a first-degree relative with PCOS may be an independent risk factor for the development of type 2 diabetes in childhood. The implication is that FDR’s of PCOS should potentially be screened more aggressively for pre-diabetic risk factors, including obesity, hypertriglyceridemia, and a pro-inflammatory state and should be considered an at-risk group in terms of efforts to prevent the development of other risk factors. Finally, the detection of impaired glucose homeostasis prior to the onset of hyperandrogenism is in agreement with the hypothesis that hyperinsulinism is a cause, rather than the result, of PCOS.

PCOS is likely the cumulative product of a number of genetic, epigenetic, environmental factors and/or familial habits [29]. PCOS may be inherited in an autosomal dominant or X-linked dominant pattern [30-32]. Genome-wide genetic and linkage studies have found associations with PCOS for many genes including fibrillin-3, PPAR-γ and IL-6, though replication has proven elusive [33-36] and development of the characteristic syndrome may occur in the absence of known mutation. In complex, heterogeneous conditions with variable presentation such as PCOS, studies of first-degree relatives of affected females may help to separate the biochemical contributions from genetic and habitual influence.

Hyperandrogenism is the consistent finding in prior studies of the adult relatives of PCOS women [22,37-39] but the data on hyperandrogenism in peripubertal FDR is scant. One study observed higher androgen levels in the later stages of puberty (Tanner 4 and 5) in PCOS-FDR subjects compared to control daughters [40], likely an expression of the normal maturation of the hypothalamic-pituitary gonadal axis.

Many have proposed that hyperinsulinism is the fundamental pathophysiological event leading to ovarian and/or adrenal production of excessive androgen [41]. Hyperandrogenism is the primary feature in the emergence of PCOS [22,42]. In our study, significant differences in the androgen precursor DHEA-S between the two groups were not detected.

Conversely, beta-cell function was impaired not only in affected PCOS probands but also in their first-degree relatives, regardless of whether PCOS or other metabolic abnormalities were yet manifest. Prior reports of insulin resistance and glucose insensitivity in first-degree relatives utilized manipulation of the less informative oral glucose tolerance test. A recent study demonstrated hyperinsulinemia and increased ovarian volumes present in PCOS daughters even prior to the onset of puberty, the hyperinsulinemia persisting throughout pubertal development. Other biochemical abnormalities of PCOS emerge only in later puberty [40], suggesting that metabolic disturbances are fundamental to establishment of permanent states of androgen excess.

Frequently-sampled IVGTT is a well-validated method of estimating insulin sensitivity and considered superior to OGTT-derived measures of insulin dynamics [43-45] as it allows determination of the glucose disposal index (GDI), a highly sensitive reflection of the capacity of pancreatic islets to compensate for lower insulin sensitivity [46]. We utilized IVGTT to assess both glucose tolerance and insulin resistance as well as the acute insulin response and glucose disposition indices that better define the beta-cell function.

Our data provides evidence of early development of insulin resistance in the peripubertal first-degree female relatives of women with PCOS. All three measures, QUICKI, HOMA-IR and GDI, demonstrated lower insulin sensitivity among PCOS first-degree relatives versus weight, Tanner, age-matched controls without family history of PCOS, diabetes mellitus and hypertension. Perturbed beta-cell dysfunction and the resulting inadequate compensation for deteriorating insulin sensitivity has been demonstrated in the daughters of PCOS-affected women prior to puberty and independently of body weight. Our findings suggest that peripubertal insulin resistance (IR) even prior to biochemical or clinically apparent androgen excess may also be an early hallmark of risk for PCOS in the genetically vulnerable peri-adolescent population as well. This emphasizes the necessity of early and ongoing biochemical monitoring of relatives of women with PCOS, affording the opportunity for both earlier diagnosis and therapeutic intervention to prevent the long-term morbidity inherent in this disorder.

## Abbreviations

PCOS: Polycystic ovarian syndrome; AIR: Acute insulin response; GDI: Glucose disposal index; QUICKI: Quantitative insulin sensitivity check index; HOMA: Homeostatic model assessment; BMI: Body mass index; IVGTT: IV glucose tolerance test; OGTT: Oral glucose tolerance test; DHEAS: Dehydroepiandrosterone sulfate.

## Competing interests

NR, AK, RP, SS, SB, AB, SM, RM, J-PB, MIH, MR, ST and DG have no competing interests.

## Authors’ contributions

DG participated in the planning and execution of the protocols performed on study subjects, as well as the preparation of the manuscript. NR, AK, RP, SS, SB, AB, SM, RM, J-PB, MIH, MR, ST participated in the execution of the protocols performed on study subjects, as well as the preparation of the manuscript. All authors read and approved the final manuscript.
